# Supplementary material for: Frequency-hopping wave engineering with metasurfaces
Source: Nat Commun. 2024 Jan 3;15:196. doi: 10.1038/s41467-023-44627-8 (PMC10764809; doi:10.1038/s41467-023-44627-8)
Supplement: Supplementary file 1 — Supplementary Information [file 41467_2023_44627_MOESM1_ESM.pdf]

Supplementary Information

Frequency-Hopping Wave Engineering with Metasurfaces

Hiroki Takeshita, Ashif Aminulloh Fathnan, Daisuke Nita, Atsuko Nagata, Shinya Sugiura and Hiroki Wakatsuchi

Supplementary Note 1: Frequency selectivity of conventional electromagnetic media
Supplementary Note 2: Frequency conversion by full wave rectification and time constants
Supplementary Note 3: Supplementary information of Fig. 2
Supplementary Note 4: Supplementary information of the simulations related to Fig. 3
Supplementary Note 5: Supplementary information of the measurements related to Fig. 3
Supplementary Note 6: Simulations and measurements for improved transmittance
Supplementary Note 7: Supplementary information of Fig. 4
Supplementary Note 8: Supplementary information of Fig. 5

Supplementary Note 1: Frequency selectivity of conventional electromagnetic media

In this Supplementary Note, we explain how metasurfaces are theoretically approximated by equivalent circuit models that are characterized by the frequency and that remain unchanged if the incoming frequency spectra are fixed. The characteristic impedance Z of a metasurface may be approximated by a simple equivalent circuit model. For instance, the conducting geometry of the slit structure shown in Fig. 2a can be represented by a parallel circuit:

$$Z(\omega) = \left(\frac{1}{j\omega L} + j\omega C\right)^{-1}, \tag{1}$$

where ω is the angular frequency associated with frequency f through $\omega = 2\pi f$, $j^2 = -1$ and L and C denote the entire inductive and capacitive components of the metasurface, respectively. Assuming a two-port network that includes the above structure, the reflection coefficient S_{11} and transmission coefficient S_{21} are obtained by

$$S_{11}(\omega) = \frac{Z(\omega) - Z_0}{Z(\omega) + Z_0}, \tag{2}$$

$$S_{21}(\omega) = \frac{Z(\omega) \cdot Z_0}{Z(\omega) + Z_0}, \tag{3}$$

where Z_0 represents the characteristic impedance of the medium next to the metasurface (vacuum in this study). Note that in Eqs. (1) to (3), the impedance of the dielectric substrate behind the conducting pattern is omitted for simplicity, although it can be represented by an additional transmission line. Moreover, the reflected energy R_{PLS} and transmitted energy T_{PLS} of the incident pulse are derived from[1]

$$R_{PLS}(\omega) = \frac{\int |b^-(\omega)|^2 d\omega}{\int |b^+(\omega)|^2 d\omega} = \frac{\int |b^+(\omega) S_{11}(\omega)\ |^2 d\omega}{\int |b^+(\omega)\ |^2 d\omega}, \tag{4}$$

$$T_{PLS}(\omega) = \frac{\int |b^+(\omega) S_{21}(\omega)\ |^2 d\omega}{\int |b^+(\omega)\ |^2 d\omega}, \tag{5}$$

where b^+ and b^- are the magnitudes of the incident and reflected waves, respectively. Therefore, Eqs. (4) to (5) indicate that R_{PLS} and T_{PLS} remain the same if the frequency

spectrum of the incident pulse is the same. Moreover, this study used pulsed sine waves, the electric field intensity E of which followed the function below:[2]

$$E(\omega) = \frac{a}{2}\left(\frac{sin(\omega - \omega_0)T_{PW}}{0.5(\omega - \omega_0)} + \frac{sin(\omega + \omega_0)T_{PW}}{0.5(\omega + \omega_0)}\right), \tag{6}$$

where ω_0 is the oscillating angular frequency and a and T_{PW} are the magnitude and pulse width of the incident pulse, respectively. In this study, the pulse width is set to 50 ns or longer in the range of a few GHz, which ensures that the pulse spectrum is almost the same as the oscillating frequency.[3] Hence, it is also clear from Eqs. (4) to (6) that under these circumstances, the scattering response essentially remains the same if the incoming frequency spectra remain unchanged.

Supplementary Note 2: Frequency conversion by full wave rectification and time constants

This Supplementary Note explains how the diode bridge and the internal circuit elements of a unit cell of a waveform-selective metasurface can effectively be approximated by the DC circuit at the bottom of Fig. 2b.[4] First, due to the diode bridge, the waveform of an incident sine wave is converted to the waveform based on a modulus of the sine function. The frequency spectra of the rectified waveform can readily be obtained by using Fourier series expansion:

$$A(t) = \frac{a_0}{2} + \sum_{n=1}^{\infty} a_{\mathrm{n}} \cos\left(\frac{2\pi n t}{T}\right) + \sum_{n=1}^{\infty} b_{\mathrm{n}} \sin\left(\frac{2\pi n t}{T}\right), \tag{7}$$

where

$$a_{\mathrm{n}} = \frac{2}{T}\int_0^T A(t)\cos\left(\frac{2\pi n t}{T}\right) dt, \tag{8}$$

$$b_{\mathrm{n}} = \frac{2}{T}\int_0^T A(t)\sin\left(\frac{2\pi n t}{T}\right) dt, \tag{9}$$

T and *n* denote the period of the incident sine wave (i.e., $T = 1/f$) and the natural number, respectively. Suppose that the rectified waveform is a modulus of a sine function, namely,

$$A(t) = \left|\sin\left(\frac{2\pi t}{T}\right)\right|, \tag{10}$$

a_{n} and b_{n} are rearranged as follows:

$$a_{\mathrm{n}} = \frac{4\cos\left(\frac{n\pi}{2}\right)^2 \cos(n\pi)}{(1-n^2)\pi}, \tag{11}$$

$$b_{\mathrm{n}} = \frac{2\big(1+\cos(n\pi)\big)\sin(n\pi)}{(n^2-1)\pi}. \tag{12}$$

According to Eqs. (11) and (12), the energy of the incident frequency *f* is converted to those of other frequencies, as seen in Table S1. This table indicates that a large portion of the

energy appears at zero frequency, which enables us to exploit the transient phenomena well known in DC circuits even if an alternating current (AC) signal comes in.

Next, as seen above, the internal circuit configuration of a unit cell of the waveform-selective metasurface is found to effectively respond to a DC signal. Under this assumption, it is well known that the transient voltage across the inductor V_L can be readily estimated by $V_\mathrm{L} = E_0 e^{-t/\tau_\mathrm{L}}$, where E_0 and t are the DC voltage applied and time, respectively, while the time constant τ_L is determined by the circuit components used[76]

$$\tau_L = L/(R_L + 2R_d). \qquad (13)$$

In Eq. (13), L and R_L are the inductance and the resistance inside the diode bridges, respectively. R_d represents the resistive component of the diodes used. Note that R_d plays an important role in determining the time constant since τ_L becomes totally different without the use of R_d.[4] Eq. (13) indicates that the waveform-selective transient responses can be readily tailored by changing the circuit values.

Table S1: Energy at each frequency component after full wave rectification.

Frequency component	Before rectification	After rectification
0	0	$(2/\pi)^2$
f	1	0
$2f$	0	$(4/3\pi)^2$
$3f$	0	0
$4f$	0	$(4/15\pi)^2$
$5f$	0	0
…	…	…

Supplementary Note 3: Supplementary information of Fig. 2

This Supplementary Note provides information related to Fig. 2. First, the design parameters and circuit values used for Fig. 2 are summarized in Figure S1, Table S2, Table S3 and Table S4. While Fig. 2c depicts a dual-band waveform-selective metasurface, conventional single-band waveform-selective metasurfaces can be designed by using the same capacitance values for C_1 and C_2, as seen in Figure S2. This figure shows that the transmittance for a 50-ns short pulse is more enhanced than that for a CW in a single-frequency band that can be adjusted by the values of C_1 and C_2. The power dependence of the dual-band waveform-selective metasurface demonstrated in Fig. 2 is shown in Figure S3. This figure indicates that the metasurface is independent of the incoming waveform with a fixed frequency if the incident power is small enough (e.g., -20 dBm), as the diodes used are not turned on yet. However, by sufficiently increasing the input power to 10 dBm, the difference between the transmittance for a short pulse and that for a CW is maximized. Additionally, Figure S3 shows that with a further increment in the input power, the transmittance for a short pulse starts decreasing because the voltage across the diodes becomes larger than the breakdown voltage. Therefore, it is important to properly design both the turn-on voltage and the breakdown voltage of diodes.

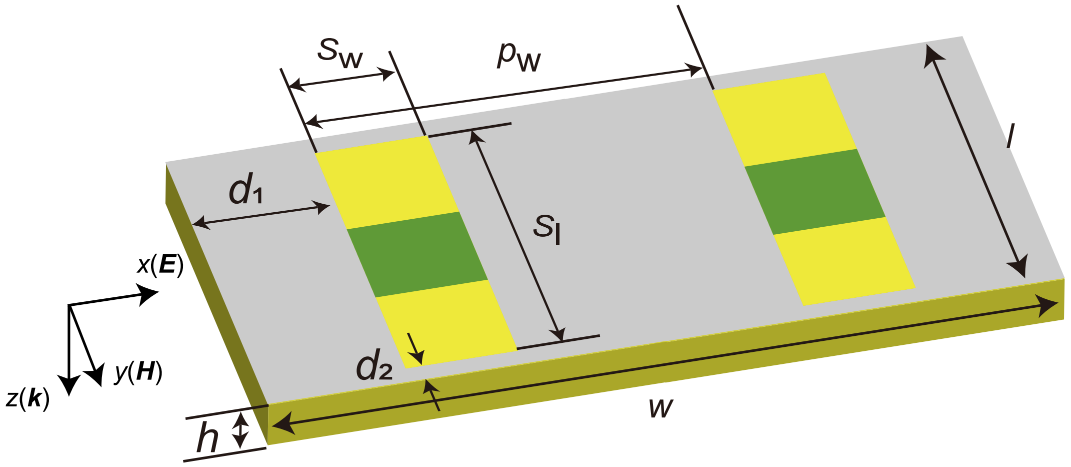

Figure S1: Supercell model used for Fig. 2. The design parameters and circuit parameters are shown in Table S2, Table S3 and Table S4. The substrate is Rogers3003.

Table S2: Design parameters used for Figure S1 (i.e., Fig. 2).

Parameter	Length [mm]
l	18
w	36
p_w	18
s_l	16
s_w	5
h	1.5
d_1	6.5
d_2	1

Table S3: Circuit values used for Figure S1 (i.e., Fig. 2). The value in parentheses represents a self-resonant frequency.

Parameter	Value
L	1 mH (2.4 MHz)
R_L	10 Ω
C_1	0.1 pF
C_2	0.6 pF

Table S4: SPICE parameters of diodes used for Figure S1 (i.e., Fig. 2).

Parameter	Value
I_{BV}	1×10^{-5} A
I_s	5×10^{-8} A
N	1.08
R_s	6 Ω
C_j	0.18 pF
M	0.5
P_B	0.65 V
P_r	2
Ev	0.69 eV

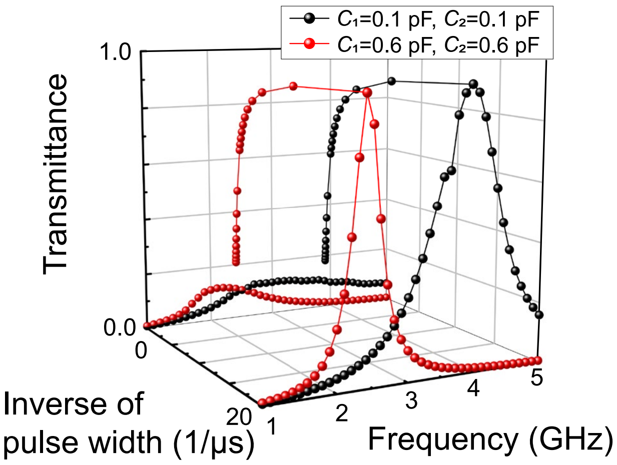

Figure S2: Transmittances of conventional single-band waveform-selective metasurfaces. C_1 and C_2 are set to the same values, as opposed to Fig. 2c, where the additional capacitances are different to achieve a dual-band operation.

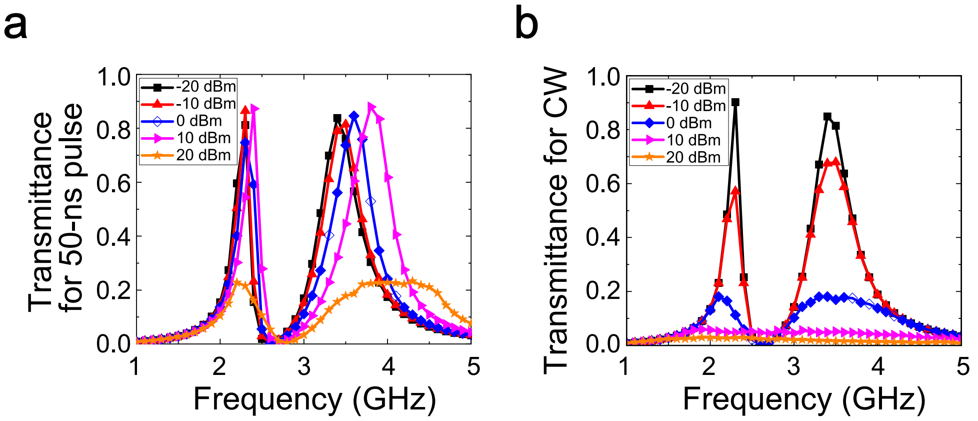

Figure S3: Power dependence of Fig. 2c. Transmittances for (a) 50-ns short pulses and (b) CWs.

Supplementary Note 4: Supplementary information of the simulations related to Fig. 3

This Supplementary Note provides information related to the numerical simulations depicted in Fig. 3. Additionally, supplementary information related to the measurement results of Fig. 3 is given in Supplementary Note 5. The design parameters and circuit values of the simulation model of Fig. 3 are the same as those used for Fig. 2 (see Figure S1, Table S2, Table S3 and Table S4). However, part of the circuit values is changed, as seen in Table S5. The frequency-domain profile of the simulation model in Fig. 3a is shown in Figure S4. As seen in this figure, the simulation model used in Fig. 3a exhibits four distinctive transmittance peaks at 2.0, 2.5, 3.3 and 4.3 GHz. However, to maximize the transient transmittance for an incoming signal whose oscillation frequency changes regularly, it may be necessary to further optimize the frequencies used. For instance, in Figure S5, 4.3 GHz (i.e., one of the four frequencies maximizing the frequency-domain transmittance) is changed between 4.1 and 4.4 GHz. The time-domain transient transmittance increases more at 4.1 GHz than at 4.3 GHz. This presumably occurs because with switching between four frequencies (2.0, 2.5, 3.3 and 4.3 GHz), which possibly leads to coupling between different cells, the voltage across diodes changes in the time domain compared to that in the frequency-domain result. Therefore, the related resistive component within the diodes is influenced, which varies the time constant and the recovery time of the entire circuit.

Table S5: Circuit values used for Fig. 3a.

Parameter	Value
L	100 μH
R_L	10 Ω
C_1	0.1 pF
C_2	0.3 pF
C_3	0.6 pF
C_4	1.1 pF

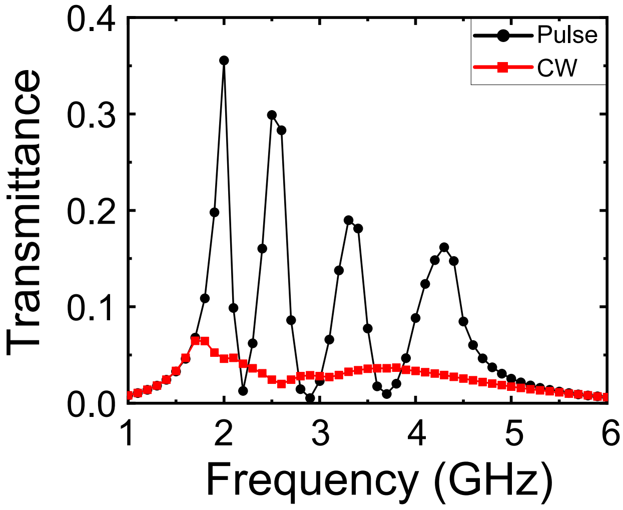

Figure S4: Frequency-domain profile of the simulation model of Fig. 3a. The pulse width and input power are set to 100 ns and 10 dBm, respectively.

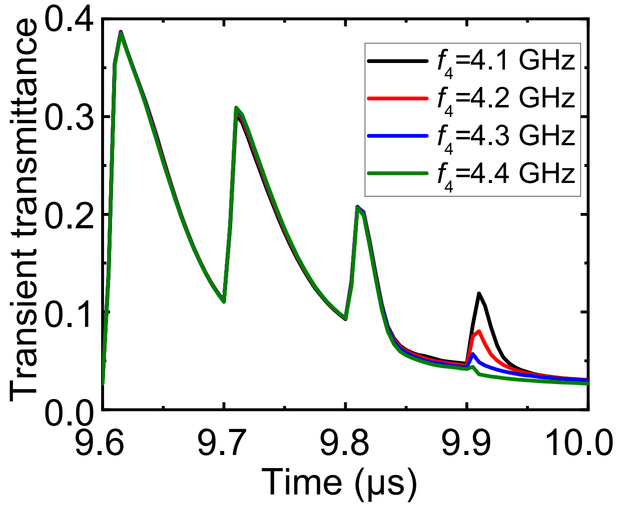

Figure S5: Simulated transient transmittance of Fig. 3a using various frequencies for f_4. Compared to Fig. 3d, the fourth frequency used is changed between 4.1 GHz and 4.4 GHz. Note that in Figure S4, the fourth transmittance peak does not appear at 4.1 GHz but at 4.3 GHz.

Regarding the recovery time to restore the inductor voltage, more information is provided in Figure S6. As seen in Figure S6a, we consider only one unit cell with periodic boundaries to readily evaluate the relationship between the recovery time and the circuit values used within the diode bridge. With an increase in the inductance from 10 μH to 10 mH, the transient transmittance for 100-ns pulses is enhanced near 4.9 GHz (Figure S6b and Figure S6c). This

is because when a large inductance value is applied, the electromotive force of the inductor is maintained for short pulses, which leads to the intrinsic resonant mechanism of the slit structure being maintained. However, the inductor voltage is found to be slowly restored to zero voltage if a large inductance is used (see Figure S6d for the result at 4.9 GHz). Therefore, there is a trade-off between the recovery time and transient transmittance level in terms of the design of the inductance value. Moreover, the frequency-domain transmittance profile is also related to the resistor deployed within the diode bridge (Figure S6e). Specifically, the transient transmittance decreases with a reduction in the resistance, as the related time constant is also increased (see Figure S6f and Supplementary Note 2). In the time domain, the smaller the resistance is, the longer the recovery time becomes (Figure S6g). Importantly, however, increasing the resistance value reduces the difference between the transient transmittance during the initial period and that in the steady state (Figure S6f); thus, the waveform-selective characteristics disappear. Therefore, there is another trade-off between the transient transmittance level and waveform-selective performance in terms of the design of the resistance value. Furthermore, to see how the recovery time and the transmittance are related to the pulse period, additional simulations are performed in Figure S6h. Here, the pulse width is fixed at 100 ns, while the duty cycle is set to either 0.25, 0.5 or 0.75 (i.e., the pulse period of 400 ns, 200 ns or 150 ns). As a result, the transient transmittance is maximized when the duty cycle is 0.25 (or the pulse period of 400 ns). This is because the inductor voltage requires a recovery time of about 300 ns to approach its original voltage value as seen in Figure S6i (see that the voltage becomes zero in approximately 300 ns after the pulse duration of 100 ns). Therefore, when the pulse period is smaller than 400 ns, the metasurface's transient transmittance cannot be maximized since the inductor voltage is not fully restored yet. For the multi-resonance case, as seen in Fig. 3d, the transient transmittance is enhanced by increasing the number of incident frequencies since it extends the pulse period

and the recovery time to restore the inductor voltage. Based on these relationships, one may need to optimize the circuit values used for waveform-selective metasurfaces.

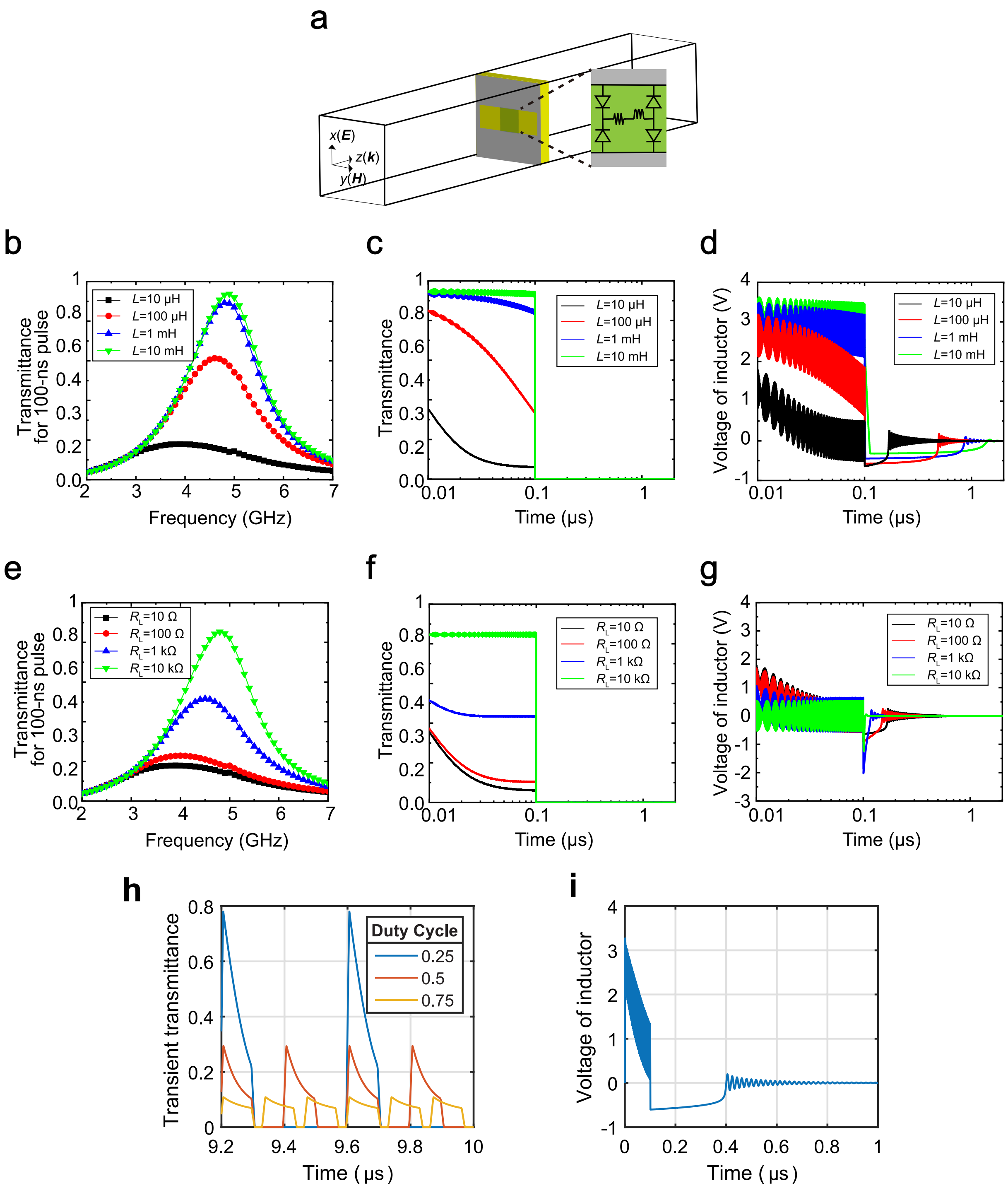

Figure S6: Relationship between circuit values and recovery time to restore inductor voltage. (a) Simulation model. Basically, the design parameters and circuit values are the same as those applied to Figure S1 (i.e., those applied to Fig. 2). (b) Frequency-domain profile with various inductances and 10-dBm input power and (c, d) corresponding time-domain profiles at 4.9 GHz. R_L is fixed at 10 Ω. (e) Frequency-domain profile with various resistances and 10-dBm input power and (f, g) corresponding time-domain profiles. L is fixed at 10 μH. In the time-domain results, incident waves are excited until 100 ns as 100-ns short pulses. (h) Time-domain profile of transient transmittance at 4.9 GHz with repeated pulses. The pulse width is fixed at 100 ns, while the duty cycle is set to either 0.25, 0.5 or 0.75. (i) Inductor voltage

during and after a 100-ns pulse. In (h) and (i), R_L and L are set to 10 Ω and 10 μH, respectively.

To better improve the overall transient transmittance of the simulation model associated with Fig. 3d, one may also optimize the oscillation duration of each frequency component. For instance, the oscillation duration is changed from 50 ns to 200 ns, as shown in Figure S7. As a result, the entire transient transmittance is found to change between 0.105 (#3 in Figure S7) and 0.140 (#1), compared to 0.138 in Fig. 3d. Note that in Figure S7, the transient transmittance for some individual frequencies is shown to be relatively large, although the average transmittance for the entire pulse period is not necessarily good (e.g., #3 in Figure S7). This is because the transient transmittance for other individual frequencies is lowered, which indicates that there is a trade-off between the transient transmittances of operating frequencies. Additionally, this transient transmittance change is strongly related to the recovery time and transient transmittance level determined by the circuit values used (see Figure S7 and the above discussion).

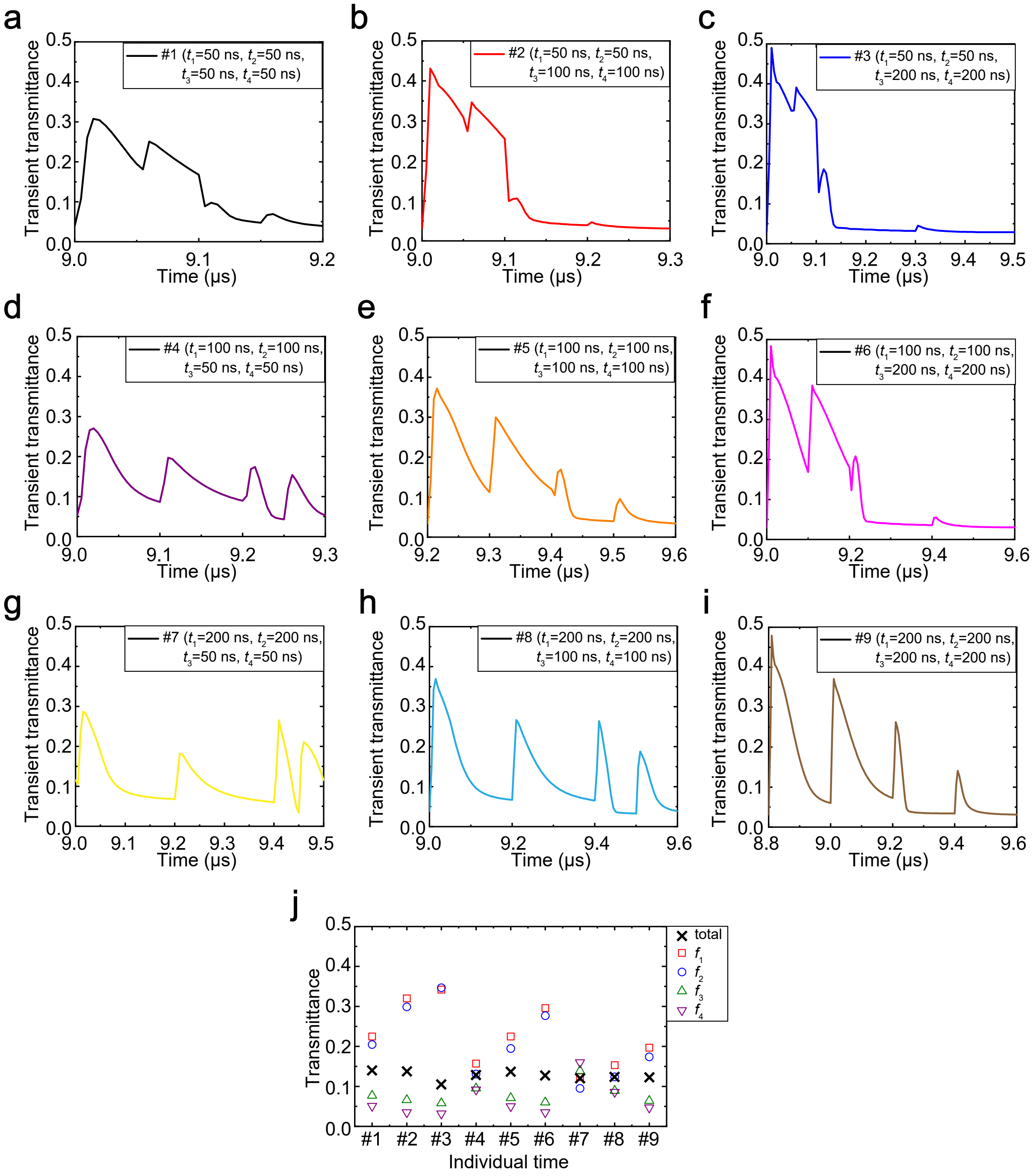

Figure S7: The transient transmittance of the simulation model depicted in Fig. 3a with various pulse duration scenarios. (a-i) From #1 to #9, the oscillation duration of each frequency is changed from 50 ns to 200 ns. (j) Average transmittance.

In contrast to Fig. 3, Fig. 4 shows transient transmittance varying in response to a particular frequency sequence. Such selectivity is not obtained by the simulation model of Fig. 3, as individual unit cells are not coupled to activate circuit structures of other cells. This is shown

in Figure S8, where the frequency sequence of an incoming wave is changed. Figure S8 shows only a minor difference among different frequency sequences, which results from small coupling between unit cells. Note that this variation is much smaller than the one seen in Fig. 4d.

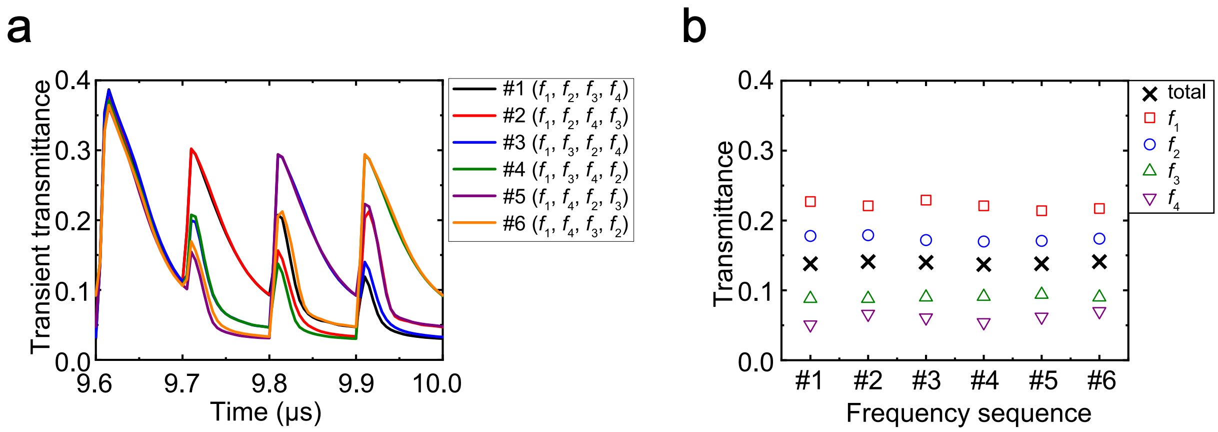

Figure S8: Transient transmittances of the simulation model drawn in Fig. 3a with different frequency sequence scenarios. (a) From #1 to #6, the frequency sequence used is changed. (b) Average transmittance.

Additionally, we numerically tested the quadband waveform-selective metasurface of Fig. 3a in free space to show that the use of our metasurfaces is not limited to one-dimensional systems. In this simulation we used a pair of standard horn antennas (Schwarzbeck BBHA 9120 D) as a transmitter and a receiver, as shown in Figure S9a and Figure S9b. The metasurface tested was composed of 6 × 6 unit cells (i.e., 3 × 3 supercells) and surrounded by a PEC wall to ensure that the incident wave transmitted through the metasurface. Other design parameters are given in Table S6. Under this circumstance, the metasurface continued to exhibit enhanced transient transmittance if the incident frequency was regularly switched, as seen in Figure S9c. This simulation result supports that the concept of our metasurfaces can be exploited in free space as well, which is important to find applications in, for instance, wireless power transfer and communications.

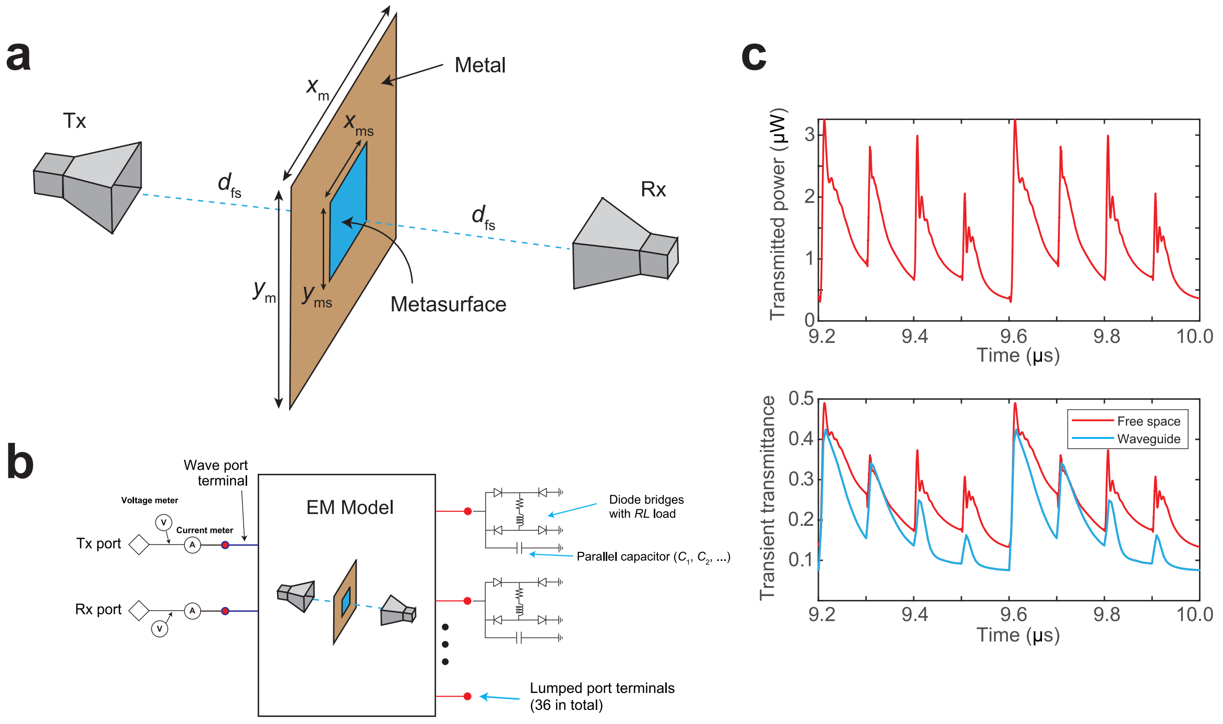

Figure S9: Simulation of the metasurface of Fig. 3a using the free-space configuration. (a) A pair of two horn antennas are used as a transmitter and a receiver in the electromagnetic simulation. The metasurface is deployed in a metallic conductor plate to narrow the metasurface area (lower the computational resources needed) while preventing the direction propagation of the signal between the transmitter and the receiver. The design parameters are shown in Table S6. (b) Circuit schematic integrating the electromagnetic simulation result. Two terminals are connected to the transmitter and receiver ports, and the remaining terminals are connected to diode bridges including inductors and resistors with parallel capacitors. (c) Simulation results of the cosimulation method. The top panel shows the transmitted power at the receiver when the transmitter generates a signal of 20 dBm. The bottom panel shows a transient transmittance that is obtained after normalization to the transmitted power when the metasurface is absent. Comparison to the waveguide simulation (the blue line and the same as Fig. 3d) shows a good agreement between both configurations, indicating the possibility of extending the proposed metasurface to free-space application scenarios.

Table S6: Design parameters used for Figure S9.

Parameter	Length [mm]
x_{ms}	108
y_{ms}	108
x_m	420
y_m	320
d_{fs}	156

Supplementary Note 5: Supplementary information of measurements related to Fig. 3

This Supplementary Note provides information related to the measurements associated with Fig. 3. The measurement sample is fabricated using the design parameters of the simulation model explained in Supplementary Note 4. However, some changes are made to deploy the lumped circuit components, as shown in Figure S10, Table S7 and Table S8. The measured frequency-domain transmittance is plotted in Figure S11, where the transmittance for short pulses is much more enhanced than that for CWs at 2.58 GHz and 3.46 GHz. These two frequencies are chosen to perform the measurements associated with Fig. 3.

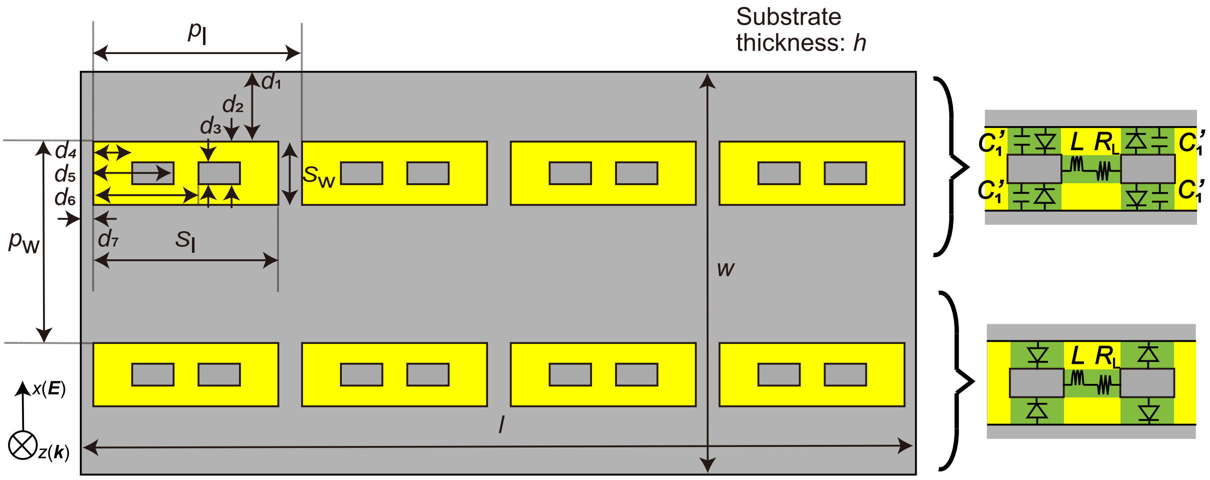

Figure S10: Design of the measurement sample shown in Fig. 3f. The design parameters and circuit values are given in Table S7 and Table S8, respectively. The substrate (yellow) is Rogers3003. In the actual measurement sample, only inductors are used inside diode bridges, as the inductors contain resistive components equal to R_L.

Table S7: Design parameters used for the unit cell model drawn in Fig. 3f and Figure S10.

Parameter	Length [mm]
l	72
w	34
p_l	18
p_w	17
s_l	16
s_w	5
h	1.52
d_1	6
d_2	3.7
d_3	1.3
d_4	4
d_5	7.5
d_6	8.5
d_7	1

Table S8: Circuit values used for the unit cell model drawn in Fig. 3f and Figure S10. The value in parentheses represents a self-resonant frequency.

Parameter	Value
L	100 μH (10 MHz)
R_L	5.5 Ω
C_1'	0.3 pF

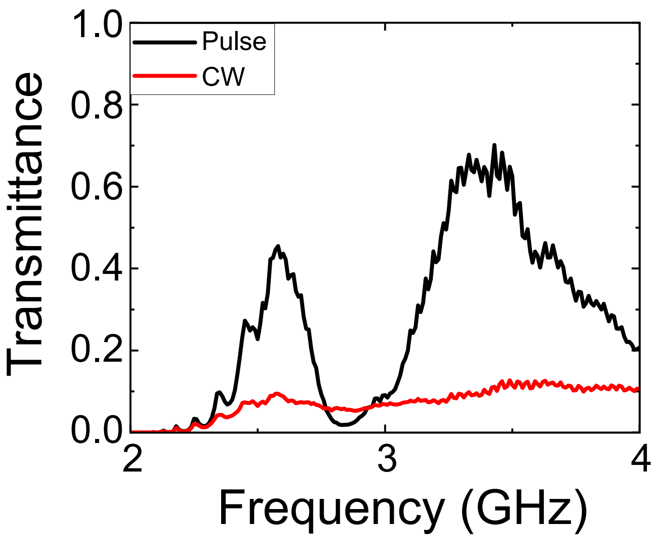

Figure S11: Frequency-domain profile of the transmittance of the measurement sample shown in Fig. 3f.

In addition, another sample is introduced in Figure S12, where the supercells are composed of three types of cells. These cells are designed to operate at different frequencies by introducing

additional capacitors, as seen in Figure S12a and Figure S12b. These design parameters are similar to those used for Figure S10, as seen in Table S9 and Table S10. The frequency-domain profiles of this measurement sample are shown in Figure S12c, which supports the operation at three different frequencies, specifically at 2.96, 3.45 and 3.92 GHz. At these frequencies, this measurement sample exhibits limited transient transmittance for CWs in the time domain, as shown in Figure S12d, while repeatedly changing the incoming frequency component increases the transient transmittance, as shown in Figure S12e. Note that the numerically derived transient transmittance for the incident wave using three frequencies, shown in Fig. 3d, is slightly larger than the measured transient transmittance, shown in Figure S12e (see the light blue curve in the top of Fig. 3d). This is because the measurement presented in Figure S12 is limited to a narrow bandwidth due to the cut-off frequencies of the rectangular waveguide used (see Figure S12c). In contrast, the simulation model associated with Fig. 3d does not have such a bandwidth limitation (Figure S4). For this reason, the simulation model is designed to have operating frequencies sufficiently far from each other, which helps avoid interference between different unit cells. Figure S12f shows the transient transmittance for different frequency sequences, which varies only between 0.065 and 0.090. Note again that this limited variation occurs because the measurement sample is not designed to couple unit cells in accordance with the states of other cells, unlike the measurement sample associated with Fig. 4.

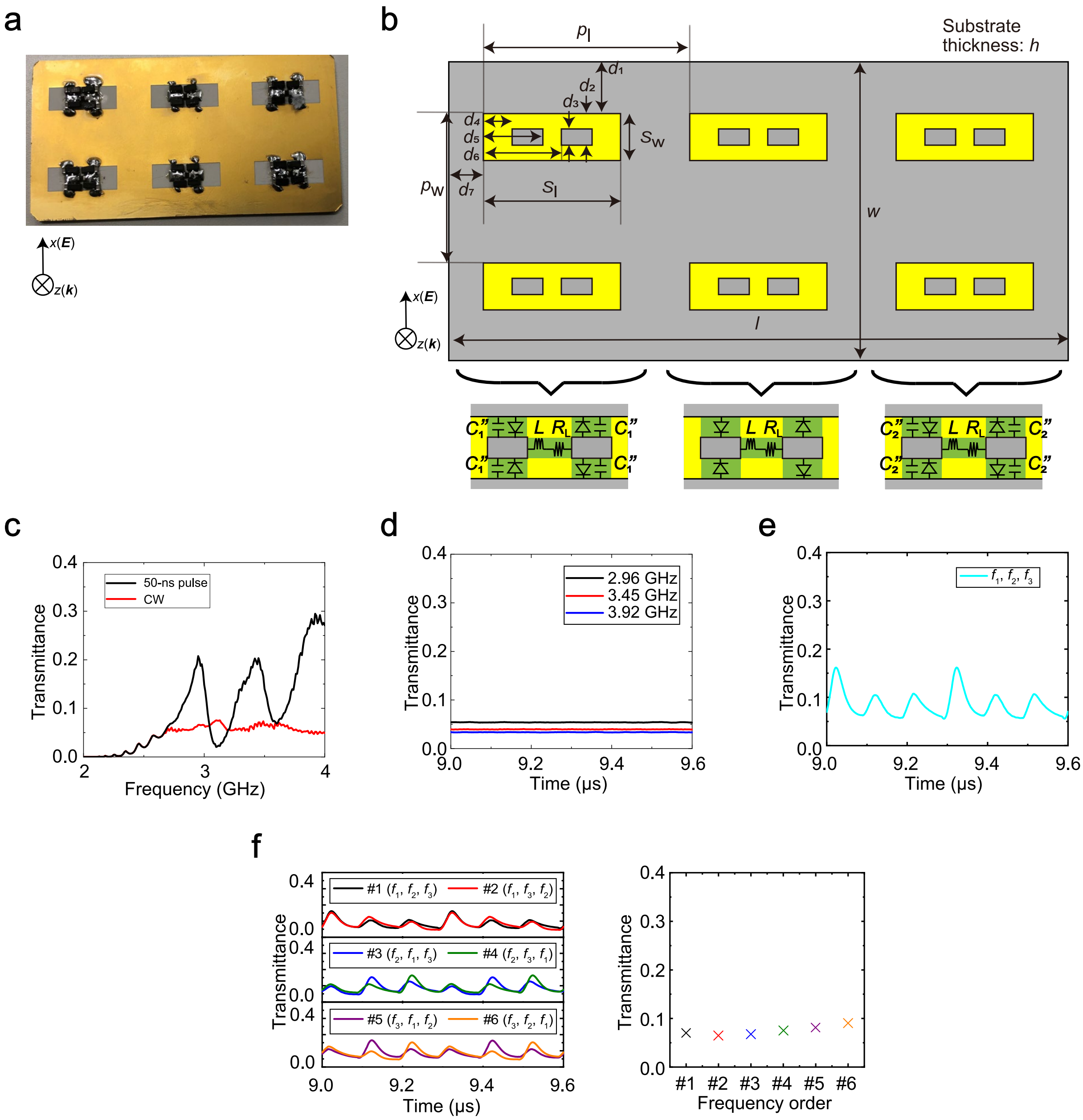

Figure S12: Measurement of the triband waveform-selective metasurface. (a, b) Measurement sample and its dimensions. The design parameters and circuit values used are shown in Table S9 and Table S10. The substrate is Rogers3003. In the actual measurement sample, only inductors are used inside diode bridges, as the inductors contain resistive components equal to R_L. (c) Frequency-domain profiles of the transmittance for 50-ns pulses and CWs. f_1 = 2.96 GHz, f_2 = 3.45 GHz and f_3 = 3.92 GHz. Time-domain profiles of the transient transmittances for (d) single-frequency cases and (e) switched-frequency case. (f) Transient transmittance for the switched-frequency case using different frequency sequences.

Table S9: Design parameters used for the measurement sample shown in Figure S12b.

Parameter	Length [mm]
l	72
w	34
p_l	24
p_w	17
s_l	16
s_w	7
h	1.52
d_1	5
d_2	1
d_3	1
d_4	4
d_5	5
d_6	8
d_7	2
d_8	1.5
d_9	2
d_{10}	3
d_{11}	2
d_{12}	1
d_{13}	1
d_{14}	0.5
d_{15}	1
d_{16}	14

Table S10: Circuit values used for the measurement sample shown in Figure S12b. The value in parentheses represents a self-resonant frequency.

Parameter	Value
L	100 μH (10 MHz)
R_L	5.5 Ω
C_1”	0.2 pF
C_2”	0.4 pF

Supplementary Note 6: Simulations and measurements for improved transmittance

Compared to the dual-band waveform-selective metasurface results (e.g., in Fig. 2c), the level of time-domain transient transmittance was relatively lowered in Fig. 3. This reduced performance results from multiple factors including loss in substrate and circuit elements. However, the transient transmittance can be improved by adjusting the Q factor. By readily demonstrating the relationship between the transient transmittance and the Q factor, we performed simplified circuit simulations where our metasurface shown in Fig. 3a was approximated by lumped circuit elements that were connected to transmission lines. First, as seen in Figure S10a, the transmitting profiles of a single slit were well represented by a capacitor C_0 paired with an inductor L_0 in parallel as well as a diode bridge including an inductor connected to a resistor in series. Here, to represent the inductive and resistive components contributed by the dielectric substrate, L_{add} and R_{add} were connected to capacitor C_0 in parallel and series, respectively. By varying the capacitors C_0 and C_1 and inductor L_0, we vary the Q factor of the resonance, as detailed in Table S7. Simulation results from the linear network analysis in Figure S10c illustrated that in the absence of any parasitic resistance (R_{add} = 0 Ω), the transmittance remained consistently high at the resonance frequency, regardless of the Q factor values. Nevertheless, when incorporating a minor amount of parasitic resistance (R_{add} = 3 Ω), the transmittance exhibits variations depending on the specific value of the Q factor. Here, the transmittance was markedly improved if the Q factor was relatively lowered as seen by the yellow line in Figure S10c. In Figure S10d, the structure was extended to have four unit cells and realize quadband operation, and similar transmittance profiles were observed. Under this situation, similar to the single resonance scenario, when there was no parasitic resistance (R_{add} = 0 Ω), the maximum transmittance consistently remained high across different Q factor values, as observed in Figure S10e. However, in the case of introducing a small parasitic resistance (R_{add} = 3 Ω), the transmittance exhibited variations

corresponding to the Q factor, as depicted in Figure S10f. In Figure S10g and Figure S10h, the incident frequency was regularly changed to obtain transient transmittance similar to Fig. 3d, in which the utilization of a lower Q factor is more favourable for a higher transient transmittance. Therefore, the transient transmittance can potentially be improved by minimizing the lossy elements of the metasurfaces and lowering the resonance's Q factor.

To validate this circuit analysis results, we have conducted an experimental investigation employing a metasurface composed of metallic bars interconnected through lumped LC resonant circuits. The schematic is shown from Figure S14a to Figure S14c. This adjustment from the original metallic slit geometry to an LC interconnection scheme allows for convenient control of the Q factor of the resonant structure to achieve higher transmittance. To test the Q factor variation, the meta-atom periodicity in the x axis is altered as seen from Figure S14a to Figure S14b. Within the rectangular waveguide, the metasurface is configured to have either three metallic rows (Figure S14d) or four metallic rows (Figure S14e). The geometrical parameters for the meta-atom as well as circuit parameters are detailed in Table S12 and Table S13. In the measurement results, we observed a reduction in the Q factor as depicted in Figure S14f (see the broadened operating bandwidths). Consequently, the lowered Q factor resulted in increasing transient transmittance, as shown from Figure S14g to Figure S14h, where the transmittance increased from 0.09 in the slit structure to 0.36 for the three-metallic-row structure, which indicates that the performance was four times greater than that of the previous slit structure design. The slit structure measurement results are detailed in Figure S12, and a similar dual-band equivalent measurement sample having frequency combination dependency is presented in Fig. 3d. Note that the average transmittance of this structure was also approximately 0.09 only.

Importantly, note that the time-varying and frequency-hopping mechanism of our structures comes from “transients in electric circuits”, namely, DC circuit mechanisms widely known to require not only imaginary part of impedance (reactance) but also the real part (resistance) potentially leading to energy loss. This fact is clarified in Eq. (13) of Supplementary Note 2, where time constant is no longer obtained if resistance is not used. Nonetheless, we showed that our new design successfully enhanced the transmittance performance by properly designing Q factors and related circuit values.

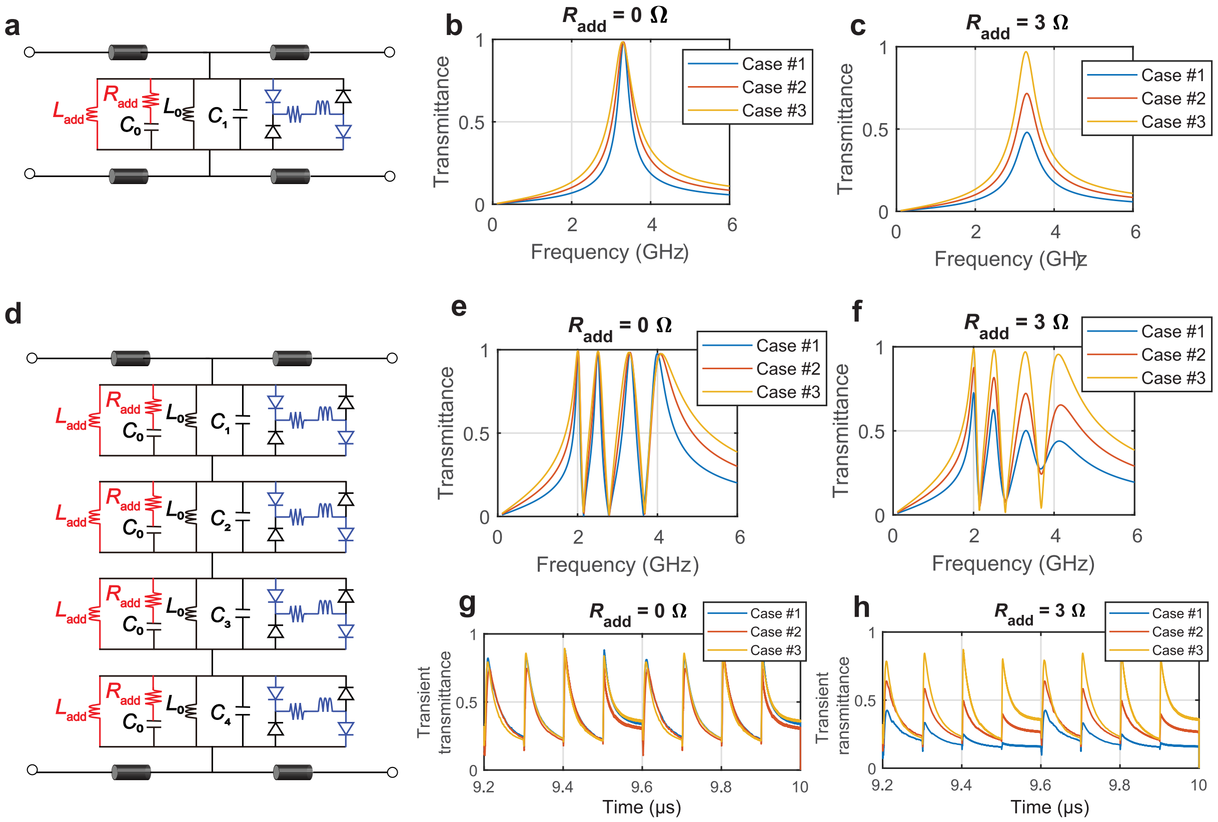

Figure S13: Simplified equivalent transmission line model to represent the metasurface of Fig. 3a for analysing the contribution of the Q factor. (a) Equivalent circuit model connected to the transmission line for the single slit scenario (one unit cell). C_0 and L_0 represent the capacitive and inductive components of the metallic slit structure. L_{add} and R_{add} represent the inductive and resistive components contributed by the dielectric substrate. The circuit parameters used for the diode and the series inductor and resistor (inside the diode bridge) are the same as those used in Fig. 3. (b, c) Simulated transmittance from linear network analysis for the circuit shown in (a) with varying Q factors while considering (b) zero parasitic resistance $R_{add} = 0$ Ω and (c) nonzero parasitic resistance $R_{add} = 3$ Ω (see Table S11 for variation of C_1). (d) Equivalent circuit model connected to the transmission line for the slit scenario (one super cell). (e, f) Simulated transmittance from linear network analysis for the circuit shown in (d) with varying Q factors while considering (e) zero parasitic resistance $R_{add} = 0$ Ω, and (f) nonzero parasitic resistance $R_{add} = 3$ Ω. (g, h) Nonlinear analysis of transient transmittance for four parallel resonance scenarios with switched frequency. Here, the input power is set to 10 dBm. f_1, f_2, f_3 and f_4 are 2.0, 2.5, 3.3 and 4.1 GHz, respectively. Simulated transient transmittance with varying Q factor while considering (g) zero parasitic resistance $R_{add} = 0$ Ω and (h) nonzero parasitic resistance $R_{add} = 3$ Ω.

Table S11: Circuit values used for Figure S13.

Single-resonance scenario				
	C_0 (pF)	L_0 (nH)	L_{add} (nH)	C_1 (pF)
Case #1	2.1	1	1.885	0.15
Case #2	1.25	2	1.885	0.15
Case #3	0.25	4	1.885	0.75

Four-resonance scenario							
	C_0 (pF)	L_0 (nH)	L_{add} (nH)	C_1 (pF)	C_2 (pF)	C_3 (pF)	C_4 (pF)
Case #1	2.1	1	1.885	0.15	1.25	3.9	7.3
Case #2	1.25	2	1.885	0.15	0.95	2.7	5
Case #3	0.25	4	1.885	0.75	1.4	2.7	4.5

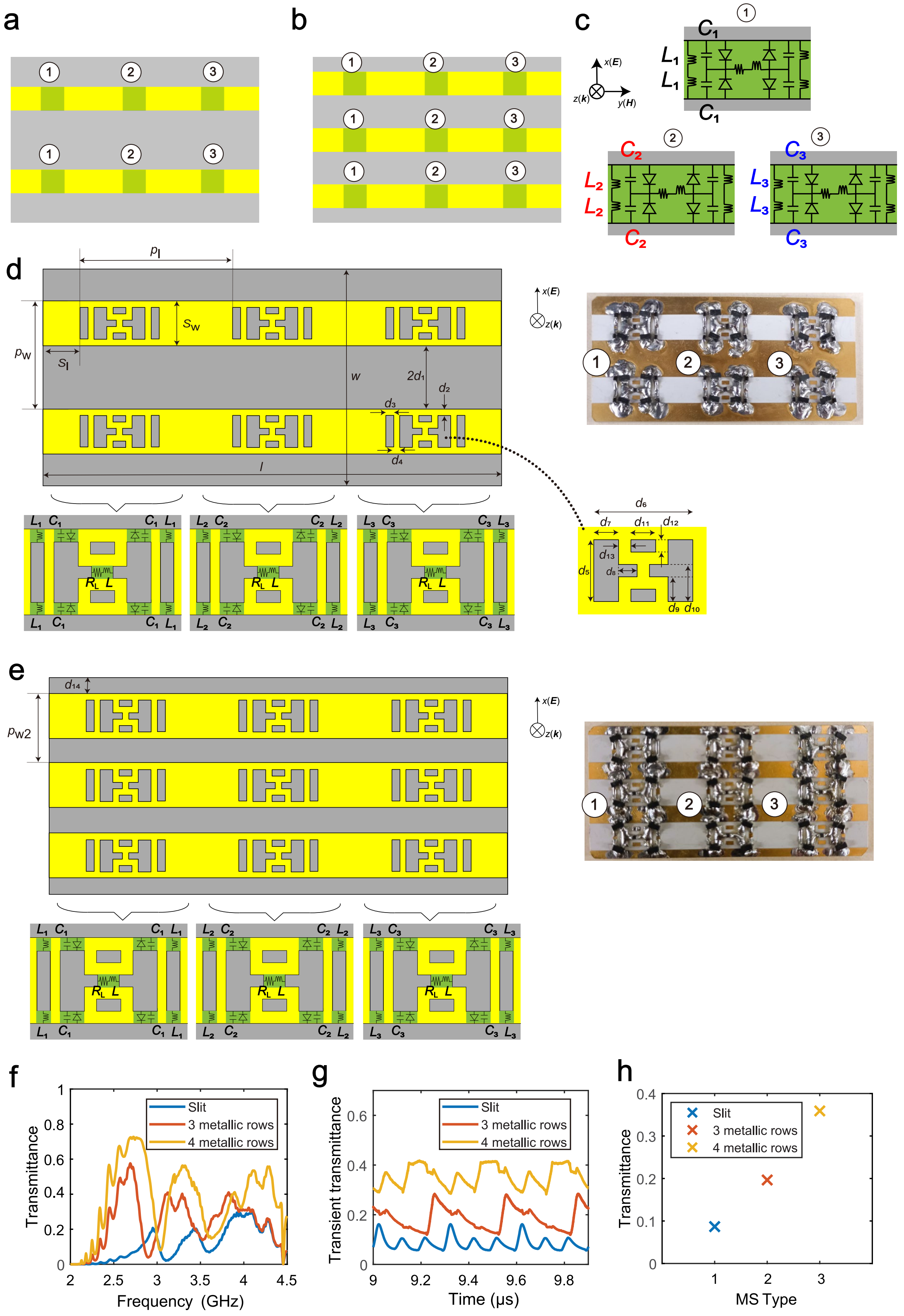
a
b
c
d
e
f
g
h
Slit
3 metallic rows
4 metallic rows
Transmittance
Frequency (GHz)
Transient transmittance
Time (μs)
MS Type

Figure S14: Experimental demonstration of metasurface configuration based on lumped LC circuit resonators for increased transmittance. (a) Schematic of the metasurface with three metallic rows inside the rectangular waveguide (6 unit cells). (b) Schematic of the metasurface with four metallic rows inside the rectangular waveguide (9 unit cells). (c) The corresponding unit cell designs. (d, e) Measurement samples and their dimensions for (d) the three-metallic-row case and (e) the four-metallic-row case. The design parameters and circuit values used are shown in Table S12 and Table S13. (f) Frequency-domain profiles of the transmittance for 50-ns pulses. The result is compared to the slit configuration of Figure S12c. (g) Measured transient transmittance for switched-frequency cases. Here, the three frequencies used, i.e., f_1, f_2 and f_3, are changed in each structure to maximize transient transmittance in three different frequency bands. Specifically, for the 3-metallic-row case, f_1, f_2 and f_3 are 2.69, 3.12 and 3.82 GHz, respectively, while for the 4-metallic-row case, f_1, f_2 and f_3 are 2.83, 3.31 and 3.91 GHz, respectively. (h) Average transmittance for entire pulse period. In these measurements, the input power is set to 10 dBm.

Table S12: Design parameters used for the measurement sample shown in Figure S14d to Figure S14e.

Parameter	Length [mm]
l	72
w	34
p_l	24
p_w	17
p_{w2}	11.33
s_l	6
s_w	7
h	1.52
d_1	5
d_2	1
d_3	1
d_4	1
d_5	5
d_6	8
d_7	2
d_8	1.5
d_9	2
d_{10}	3
d_{11}	2
d_{12}	1
d_{13}	1
d_{14}	2.167

Table S13: Circuit values used for the measurement sample shown in Figure S14d to Figure S14e. The number in parentheses represents a self-resonant frequency.

Parameter	Value
L	1 mH (2.4 MHz)
R_L	10 kΩ
C_1	0.2 pF
C_2	0.1 pF
C_3	0.6 pF
L_1	3.3 nH
L_2	2.2 nH
L_3	2.2 nH

Supplementary Note 7: Supplementary information of Fig. 4

The concept of the measurement sample demonstrated in Fig. 4 can be simplified by using two circuit systems, as shown in Figure S15a. In this simplified concept, the left circuit is used to control the right circuit. Based on this concept, the measurement sample depicted in Figure S15b, Figure S15c and Figure S15d that uses supercells composed of only two types of unit cells is fabricated. Basically, these design parameters are the same as those used for the sample associated with Fig. 4 (specifically, see Table S14 and Table S15). Under these circumstances, the measurement sample operates at 2.7 and 3.6 GHz, as shown in Figure S15e. By using these two frequencies, the measurement sample strongly transmits an incoming frequency if the frequency component is changed from 2.7 GHz to 3.6 GHz with 20-dBm input power, as seen in Figure S15f and Figure S15g. This is because the unit cells working at 2.7 GHz are used to vary the JFETs included in the cells operating at 3.6 GHz. Since only two frequencies are used here, the measurement sample exhibits the same transient transmittance in the steady state, as shown in Figure S15h and Figure S15i.

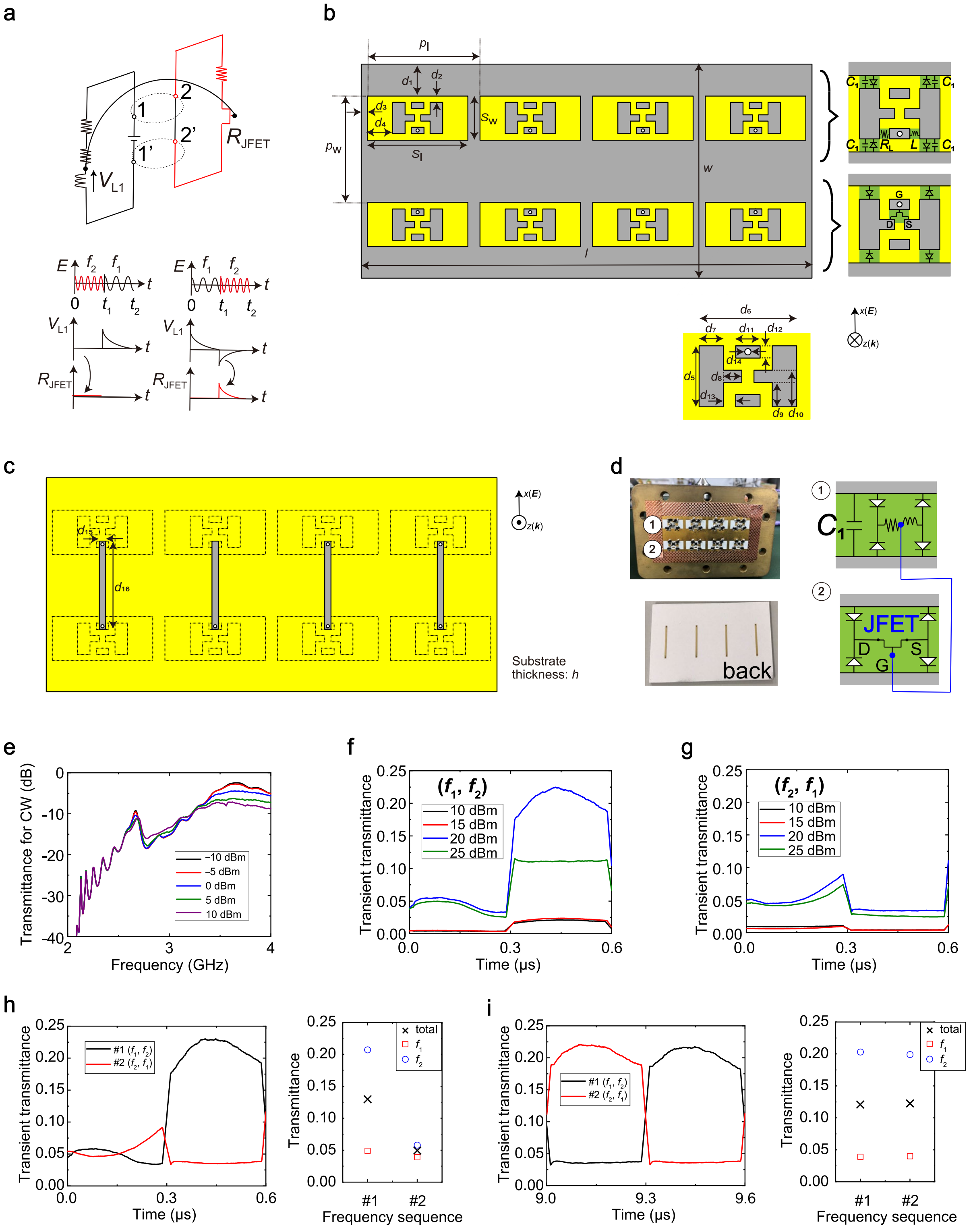

Figure S15: Demonstration of the simplified concept of Fig. 4. (a) Simplified circuit system. Compared to Fig. 4b, only two circuit configurations (i.e., two frequencies) are considered. V_{L1} and R_{JFET} vary in accordance with the incoming frequency. (b) Front and (c) back designs of (d) the measurement sample. The design parameters and circuit values are given in Table S14, Table S15 and Table S16. The substrate is Rogers3003. (e) Frequency-domain profiles of transmittance with various input powers. (f, g) Time-domain profiles of transient transmittance using different frequency sequences. f_1 = 2.7 GHz and f_2 = 3.6 GHz. The time-

domain transient transmittances (h) during the initial period and (i) in the steady state with 20-dBm input power.

Table S14: Design parameters used for the measurement sample shown in Figure S15b to Figure S15d.

Parameter	Length [mm]
l	72
w	34
p_l	18
p_w	17
s_l	16
s_w	7
h	1.52
d_1	5
d_2	1
d_3	1
d_4	4
d_5	5
d_6	8
d_7	2
d_8	1.5
d_9	2
d_{10}	3
d_{11}	2
d_{12}	1
d_{13}	1
d_{14}	0.5
d_{15}	1
d_{16}	14

Table S15: Circuit values used for the measurement sample shown in Figure S15b to Figure S15d. The number in parentheses represents a self-resonant frequency.

Parameter	Value
L	1 mH (2.4 MHz)
R_L	10 kΩ
C_1'	0.2 pF

Table S16: SPICE parameters used for the JFETs of the measurement sample shown in Figure S15b to Figure S15d. These SPICE parameters are identical to those of a commercial product provided by Toshiba (2SK880-Y).

Parameter	Value
I_{DSS}	1.2 to 3.0 mA
R_L	-0.2 to -1.5 V
C_{iss}	13 pF
C_{rss}	3 pF

The design parameters and circuit values used for Fig. 4c are provided in Figure S16, Table S17, Table S18 and Table S19. The power dependence of the frequency-domain and time-domain profiles of the sample can be seen in Figure S17. As shown in this figure, the measurement sample is designed to operate near 2.5, 3.3 and 3.9 GHz and that its optimum power level is approximately 20 dBm. In addition, Figure S18 shows how the time-domain profiles change by adjusting the oscillation frequencies. Moreover, Figure S19 provides additional information on the transmittance shown on the right of Fig. 4d.

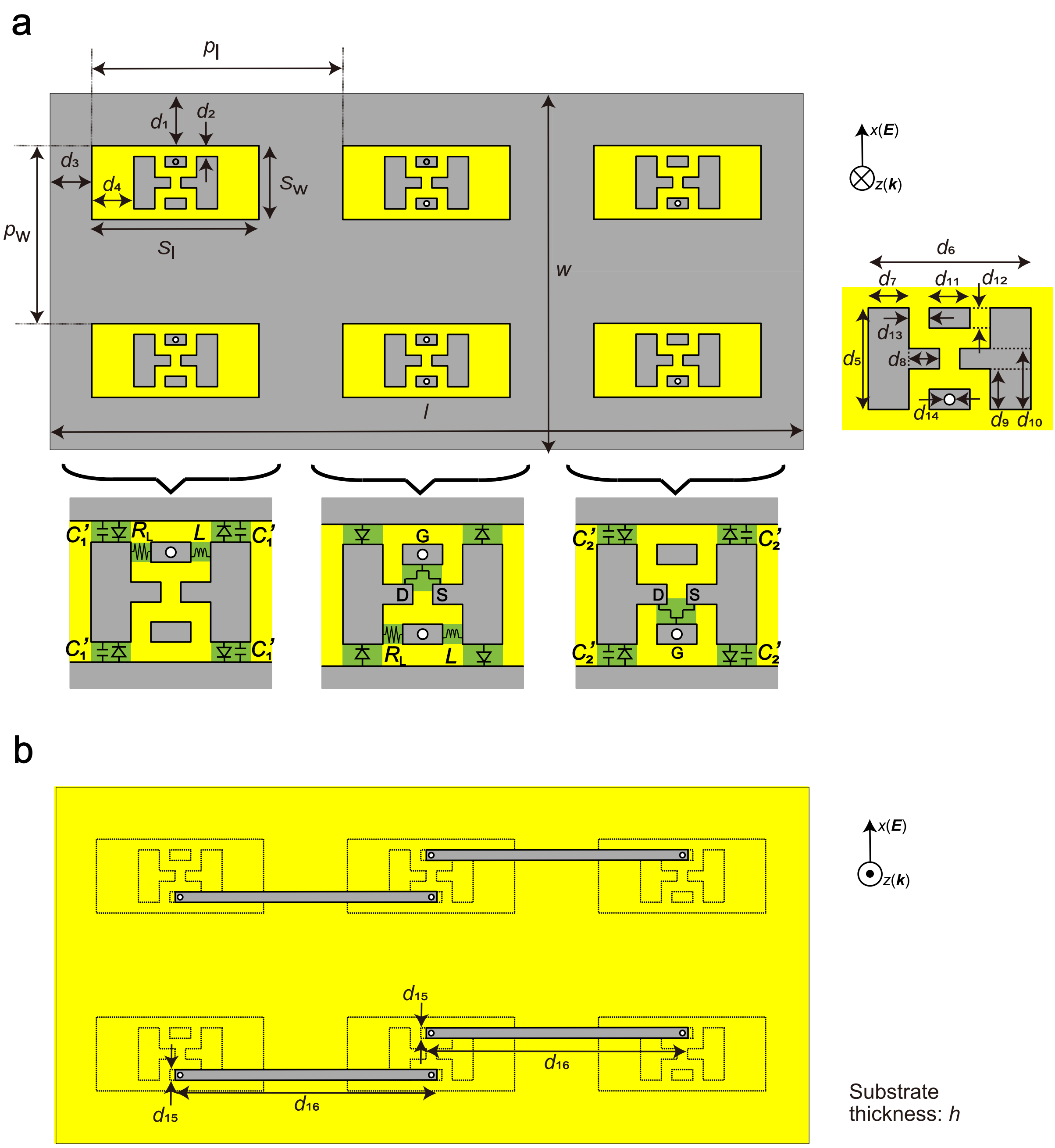

Figure S16: Design of the measurement sample presented in Fig. 4c. (a) Front and (b) back designs. The design parameters and circuit values are given in Table S17, Table S18 and Table S19. The substrate is Rogers3003.

Table S17: Design parameters used for the measurement sample shown in Figure S16 (i.e., Fig. 4c).

Parameter	Length [mm]
l	72
w	34
p_l	24
p_w	17
S_l	16
S_w	7
h	1.52
d_1	5
d_2	1
d_3	4
d_4	4
d_5	5
d_6	8
d_7	2
d_8	1.5
d_9	2
d_{10}	3
d_{11}	2
d_{12}	1
d_{13}	1
d_{14}	0.5
d_{15}	1
d_{16}	25

Table S18: Circuit values used for the measurement sample shown in Figure S16 (i.e., Fig. 4c). The number in parentheses represents a self-resonant frequency.

Parameter	Value
L	1 mH (2.4 MHz)
R_L	10 kΩ
C_1’	0.2 pF
C_2’	0.4 pF

Table S19: SPICE parameters used for the JFETs of the measurement sample shown in Figure S16 (i.e., Fig. 4c). These SPICE parameters are identical to those of a commercial product provided by Toshiba (2SK880-BL).

Parameter	Value
I_{DSS}	6.0 to 14.0 mA
R_L	-0.2 to -1.5 V
C_{iss}	13 pF
C_{rss}	3 pF

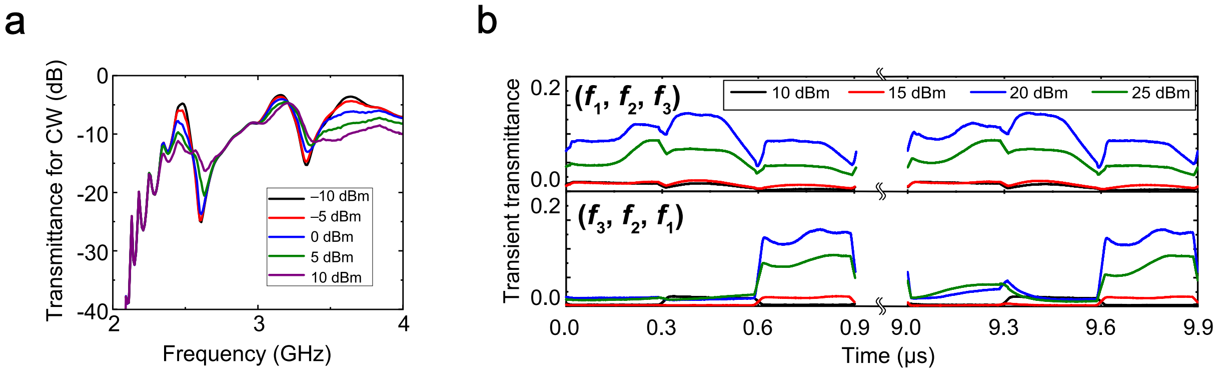

Figure S17: Power dependence of the measurement sample shown in Fig. 4c. (a) Frequency-domain and (b) time-domain profiles. In (b), f_1, f_2 and f_3 were set to 3.3, 3.9 and 2.5 GHz, respectively.

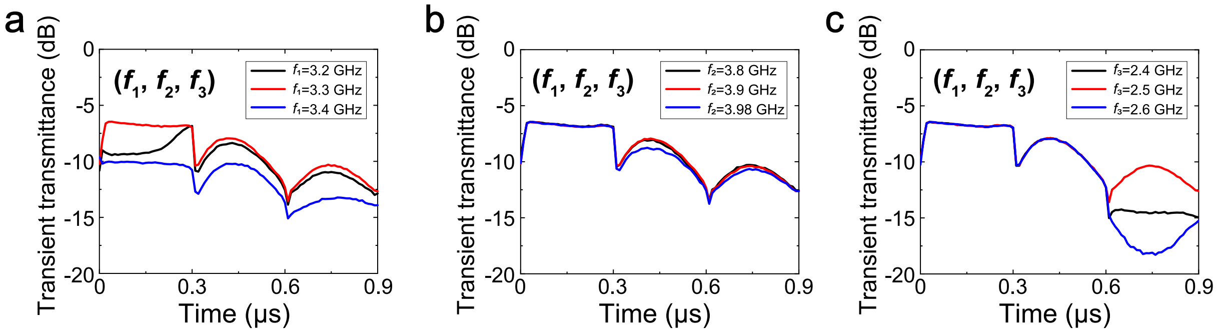

Figure S18: Time-domain profiles of the measurement sample demonstrated in Fig. 4c with adjusted oscillation frequencies. The results with various (a) f_1, (b) f_2 and (c) f_3 values. As the default values, f_1, f_2 and f_3 are set to 3.3, 3.9 and 2.5 GHz, respectively.

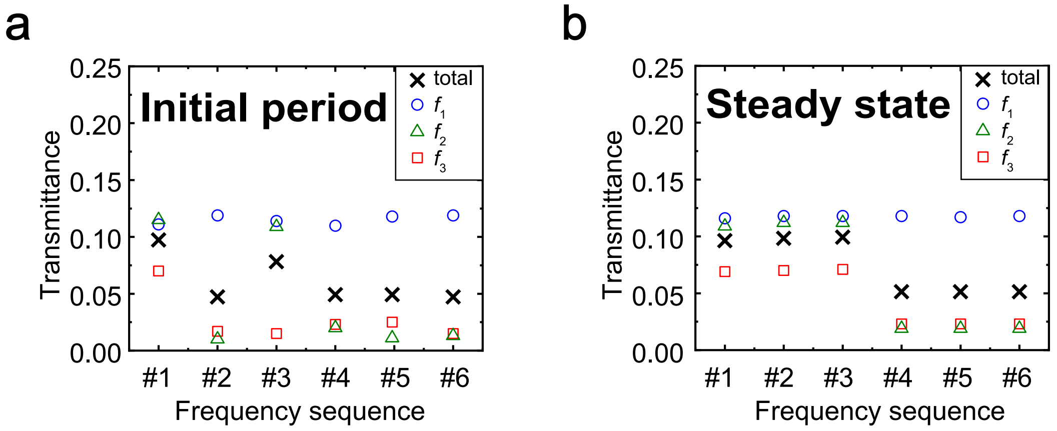

Figure S19: Average transmittance of the measurement sample demonstrated in Fig. 4c for each frequency. (a) The transmittance during the initial period. (b) The transmittance in the steady state. f_1, f_2 and f_3 are set to 3.3, 3.9 and 2.5 GHz, respectively.

As a design strategy for the preferable sequence of frequencies, one may change connection lines among unit cells. As depicted in Figure S20a, even with the same unit cell alignment including three different operating frequencies, connection lines can be arranged to determine the frequency sequence that maximizes the transmittance. In this instance, by altering the physical connection between unit cells, the preferable sequence is changed from $f_1f_2f_3$ to $f_1f_3f_2$. However, some conducting geometry designs may show nonnegligible coupling between unit cells, which lowers the transmittance performance. In this case, alternatively the capacitance values can be changed as shown in Figure S20b, where the capacitance values used for two unit cells are swapped to change the preferable frequency sequence from $f_1f_2f_3$ to $f_1f_3f_2$.

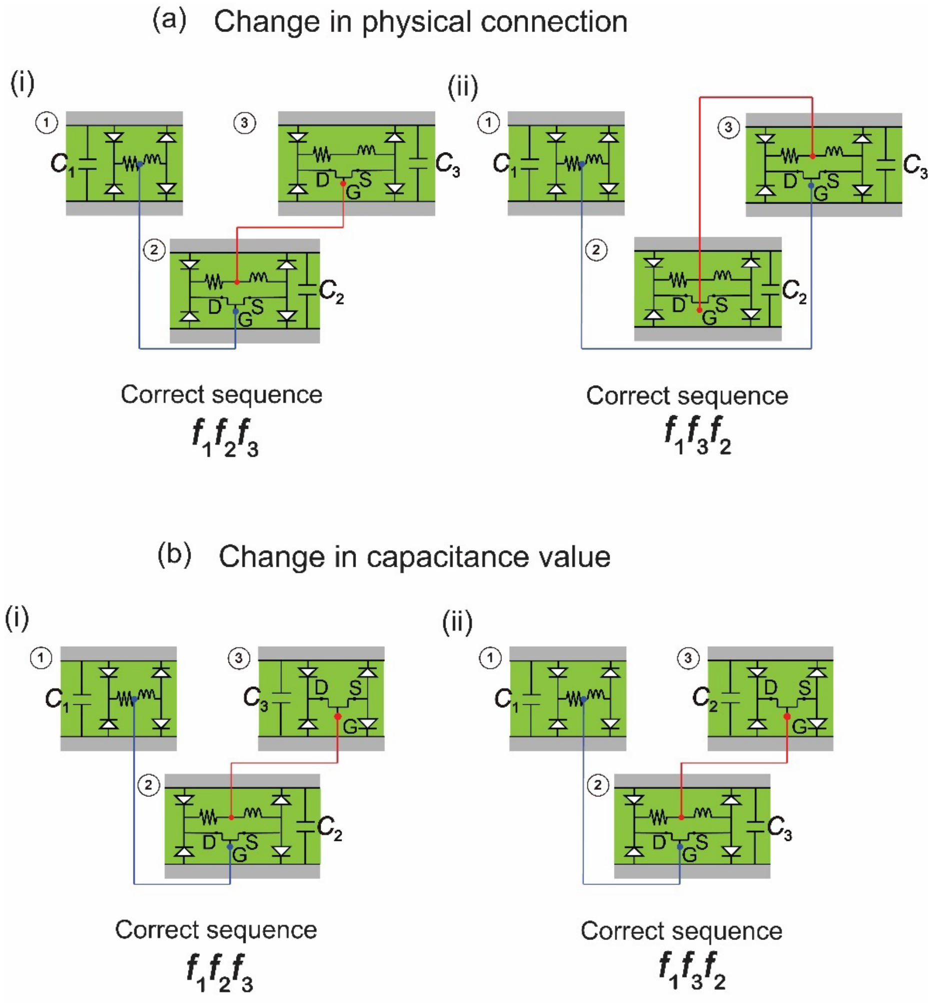

Figure S20: Design strategy for the preferable sequence of frequencies within a supercell configuration. (a) The same supercell with different physical connections. (b) The same supercell with different capacitance values.

Supplementary Note 8: Supplementary information of Fig. 5

As an additional proof-of-concept experiment, we transmit a binary image using the proposed metasurface configuration and report the obtained results in Figure S21. BPSK modulation with a frequency-hopping carrier is used as explained in the "Modulation Method" of the Methods section (see the diagram block shown in Fig. 5a). Since AWGN is added to the received signals, it is possible to evaluate the retrieved image with variation in the signal-to-noise ratio (S/N). Figure S21 illustrates the differentiation between two frequency sequences as well as the resulting image quality under varying S/N conditions. The retrieved image demonstrates enhanced clarity with fewer errors when the frequency sequence aligns with the embedded metasurface sequence (f_1, f_2 and f_3), and this distinction persists even at higher S/N values (e.g., S/N = 0 dB). These findings align with the BER versus S/N graph presented in Fig. 5c. Importantly, in this communication scenario, both the transmitter and receiver possess knowledge of the frequency sequence used for frequency hopping. Thus, the differentiation arises based on the consistency or inconsistency of the frequency sequence with the one determined or embedded within the metasurface. We also note that the difference between the frequency sequences at the same S/N would be clearer if the gap between the transient transmittances for these frequency sequences are further improved, which leads to an increase in the difference in the BER characteristics in Fig. 5c.

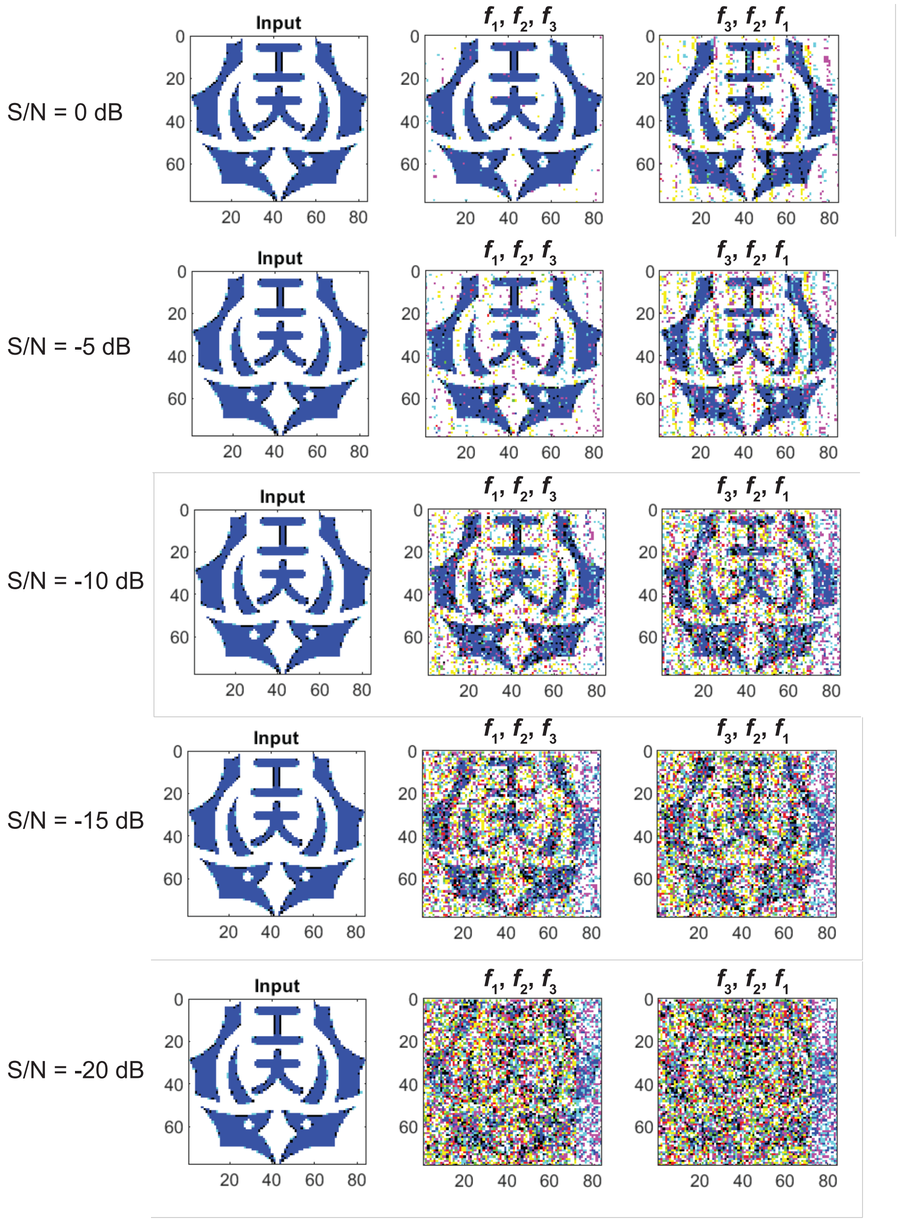

Figure S21: Example of data transmission (logo mark of Nagoya Institute of Technology) using the diagram block shown in Fig. 5a. The S/N is varied from -20 to 0 dB. In these measurements, the input power is set to 20 dBm. Two frequency sequences are used for the carrier, similar to the scenario explained in Fig. 5c.

In Fig. 5i, the transmitted energy reduction is calculated including all the frequencies used. The contribution of each frequency component is clarified in Figure S22. Note that the transmitted energy of each frequency component is still compared to that of the signal using sequence #1, as shown in Fig. 5i. Under this circumstance, the transmitted energy of the interference signal increases linearly with I/S. When I/S equals 0 dB (i.e., when the interference magnitude equals the signal magnitude), the transmitted energy of f_5 slightly exceeds the transmitted energies of f_1, f_2 and f_3. This is due to the continuous waveform nature of f_5 compared to the pulse-based signals in f_1, f_2 and f_3. Nevertheless, as observed in Fig. 5i, the sum of the energies of f_1, f_2 and f_3 is still larger than that of f_4 and f_5. Particularly, the interference energy exceeds the signal energy of frequency sequence #6 but not the signal energy of frequency sequence #1. This indicates that the presence of adjacent interference results in minimal reduction (less than 3 dB) in the signal energy, ensuring that communication performance can be maintained. However, the influence on the communication performance may vary depending on the level of the interference signal.

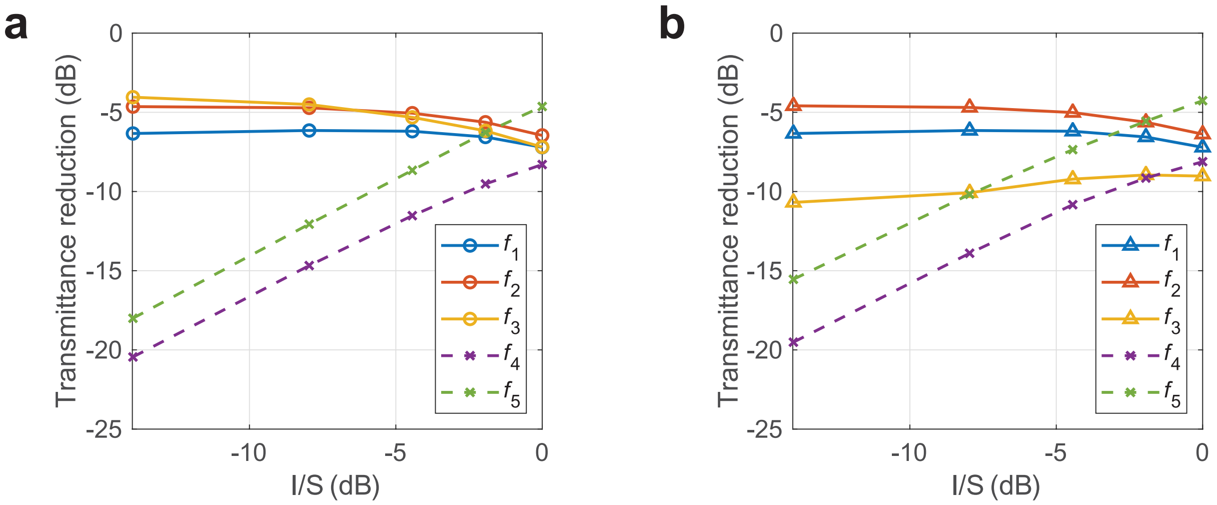

Figure S22: Contribution of each frequency used in Fig. 5i. (a, b) Results for frequency sequences (a) #1 and (b) #6. The result of the interference signal used in Fig. 5i is calculated by the sum of f_4 and f_5 in (a).

References

1. Nakasha, T., Phang, S. & Wakatsuchi, H. Pseudo-Waveform-selective metasurfaces and their limited performance. *Adv Theory Simulations* **4**, 2000187 (2021).

2. Wakatsuchi, H. Time-domain filtering of metasurfaces. *Sci Rep* **5**, 16737 (2015).

3. Wakatsuchi, H., Long, J. & Sievenpiper, D. F. Waveform selective surfaces. *Adv Funct Mater* **29**, 1806386 (2019).

4. Asano, K., Nakasha, T. & Wakatsuchi, H. Simplified equivalent circuit approach for designing time-domain responses of waveform-selective metasurfaces. *Appl Phys Lett* **116**, 171603 (2020).
